# Predictors of In-Hospital Mortality Among Hospitalized Colistin-Treated Patients at a Tertiary Urology–Nephrology Referral Center

**DOI:** 10.3390/antibiotics15090866

**Published:** 2026-09-04

**Authors:** Viorel Dragos Radu, Ana-Maria Raluca Pauna, Rodica Radu, Liliana Mititelu-Tartau, Alin Mihai Vasilescu, Theodor Florin Pantilimonescu, Adriana Grigoraș, Laura Florea, Marius Constantin Moraru, Liliana Chelaru, Liviu Ciprian Gavril, Roxana Florentina Gavril, Beatrice Rozalina Buca, Angy Abu Koush, Irina Luanda Mititiuc

**Affiliations:** “Grigore T. Popa” University of Medicine and Pharmacy, Universitatii No. 16 Street, 700115 Iasi, Romania; viorel.radu@umfiasi.ro (V.D.R.); rodica.radu@umfiasi.ro (R.R.); liliana.tartau@umfiasi.ro (L.M.-T.); alin.vasilescu@umfiasi.ro (A.M.V.); pantilimonescu.theodor-florin@d.umfiasi.ro (T.F.P.); adriana.grigoras@umfiasi.ro (A.G.); laura.florea@umfiasi.ro (L.F.); marius.moraru@umfiasi.ro (M.C.M.); liliana.chelaru@umfiasi.ro (L.C.); liviu.gavril@umfiasi.ro (L.C.G.); roxana-florentina.sufaru@umfiasi.ro (R.F.G.); beatriceroza@yahoo.com (B.R.B.); maierean_angy@yahoo.com (A.A.K.); luanda.mititiuc@umfiasi.ro (I.L.M.)

**Keywords:** colistin, polymyxin, in-hospital mortality, predictors, multidrug-resistant Gram-negative bacteria, sepsis, acute kidney injury, retrospective cohort

## Abstract

**Background/Objectives**: Colistin is a last-resort agent against multidrug-resistant Gram-negative infections and is administered predominantly to complex, comorbid inpatients in whom mortality is high. We aimed to identify independent baseline predictors of in-hospital mortality in colistin-treated patients and to characterize temporal changes in mortality over 15 years. **Methods**: We conducted a retrospective cohort study of 1225 consecutive patients receiving systemic colistin between November 2009 and November 2024 at Parhon Hospital, a tertiary urology–nephrology center in Iași, Romania; independent predictors of death were identified using multivariable logistic regression restricted to variables fixed at the time of colistin initiation, reported as adjusted odds ratios (aOR) with 95% confidence intervals (CI). **Results**: Patients were predominantly male (60.2%), with a mean age of 65.1 ± 14.4 years; 85.6% were admitted to urology or nephrology. In-hospital mortality was 27.6%. Mortality was independently associated with acute kidney injury (aOR 3.72, 95% CI 2.76–5.01), sepsis (aOR 2.79, 95% CI 2.11–3.70), emergency admission (aOR 1.91, 95% CI 1.40–2.61), Charlson comorbidity index (aOR 1.13 per point, 95% CI 1.05–1.22), and older age (aOR 1.03 per year, 95% CI 1.02–1.04); sex was not associated. Crude mortality increased across the study period (odds ratio 1.16 per year, 95% CI 1.13–1.20) and remained elevated after adjustment for case-mix (adjusted odds ratio 1.13 per year, 95% CI 1.09–1.17). **Conclusions**: Acute kidney injury, sepsis, emergency admission, comorbidity burden, and age were independently associated with in-hospital death. Mortality rose progressively over 15 years, only partly explained by an increasingly severe case-mix, supporting early risk stratification of colistin recipients.

## 1. Introduction

Antimicrobial resistance in Gram-negative bacteria has become one of the defining therapeutic challenges of contemporary hospital medicine. In its 2024 Bacterial Priority Pathogens List, the World Health Organization retained carbapenem-resistant *Klebsiella pneumoniae*, *Acinetobacter baumannii*, and other carbapenem-resistant Enterobacterales in the critical priority category, with carbapenem-resistant *K. pneumoniae* ranked as the highest-priority pathogen overall [1]. These organisms combine high transmissibility with a scarcity of effective agents, and infections caused by them are associated with substantial morbidity, prolonged hospitalization, and elevated mortality, particularly among critically ill and comorbid inpatients [2,3,4,5]. This burden is unevenly distributed across Europe and falls disproportionately on its eastern and south-eastern regions. In European surveillance data, national percentages of carbapenem resistance among invasive *K. pneumoniae* isolates vary widely across EU/EEA countries, and Romania has consistently ranked among the most affected member states, with approximately half of invasive *K. pneumoniae* isolates resistant to carbapenems in recent surveillance years [6,7]. In this epidemiological context, colistin has retained a substantially broader therapeutic role in Romanian hospitals than in most Western European settings, and locally derived prognostic data are correspondingly relevant [8,9].

Against this backdrop of therapeutic scarcity, the polymyxins—colistin (polymyxin E) and polymyxin B—have been reintroduced into routine practice as last-resort agents. Colistin, a polypeptide antibiotic first described around 1950, was largely abandoned in the 1970s because of nephrotoxicity once safer agents became available, and has since been readopted as a salvage therapy against multidrug-resistant (MDR), extensively drug-resistant (XDR), and pandrug-resistant Gram-negative organisms [6,7,8]. The growing use of colistin has been driven by rising rates of carbapenem resistance [9,10]. In this context, colistin has been widely used to treat multidrug-resistant urinary tract infections. Its clinical value, however, remains constrained by a narrow therapeutic index, with nephrotoxicity the principal dose-limiting toxicity. Reported rates of colistin-associated acute kidney injury range widely, from approximately 21% to 75% across cohorts, with pooled estimates near half of treated patients [11,12,13,14,15], reflecting differences in acute kidney injury definitions, dosing appropriateness, baseline renal function, and concomitant nephrotoxic exposure.

The populations in which colistin is deployed are, by definition, clinically complex: elderly, multimorbid, and frequently already burdened by renal impairment. Reported outcomes are correspondingly poor. In cohorts of patients treated for carbapenem-resistant Gram-negative infection, all-cause 28-day mortality has approached 44%, with intensive-care mortality near 50% [16,17], although figures vary with case-mix, formulation, and dosing strategy [18,19,20,21]. A recurring set of prognostic factors has emerged across studies: older age, higher Charlson comorbidity burden, elevated baseline serum creatinine, and colistin-associated nephrotoxicity have each been independently associated with in-hospital mortality [22,23]. Yet much of the available evidence derives from small, single-unit, or predominantly intensive-care cohorts reporting short-term mortality, which limits generalizability to the broader inpatient population receiving colistin outside the intensive-care setting.

This evidence gap is especially relevant in tertiary urology and nephrology centers, where colistin is administered to a patient population that is simultaneously infection-prone—owing to instrumentation, obstructive uropathy, and immunosuppression after transplantation—and renally vulnerable, and in whom the drug’s nephrotoxic liability is therefore of particular concern [24,25,26,27,28,29]. Large, long-term data characterizing which patients are at greatest risk of death in this specific setting remain scarce. Robust identification of baseline predictors, anchored to the clinical state at the time of colistin exposure rather than to terminal events, would support earlier risk stratification and more judicious use of a toxic last-line agent.

Accordingly, we conducted a 15-year retrospective cohort study of consecutive hospitalizations receiving systemic colistin at a tertiary urology–nephrology center. The aim of this study was to identify independent baseline predictors of in-hospital mortality among colistin-treated patients and to characterize temporal changes in mortality over the 15-year study period, in order to inform early risk stratification in this high-risk population.

## 2. Results

### 2.1. Cohort Characteristics

A total of 1409 hospitalizations receiving systemic colistin between November 2009 and November 2024 were identified, corresponding to 1225 unique patients; the first colistin-treated admission of each patient was analyzed. Patients were predominantly male (737/1225; 60.2%), with a mean age of 65.1 ± 14.4 years, and the majority were admitted to a urology or nephrology ward (1049/1225; 85.6%). Admission occurred via the emergency route in 61.6% of cases. The median Charlson comorbidity index was 3 (interquartile range [IQR] 2–4). Chronic kidney disease or end-stage renal disease was present in 46.4% of patients, acute kidney injury in 25.9%, diabetes mellitus in 28.1%, and sepsis in 40.0%. The median colistin course was 4 days (IQR 2–7), and the median length of hospital stay was 13 days (IQR 8–21). The baseline characteristics of the cohort, stratified by survival status, are summarized in Table 1.

The corresponding median cumulative quantity was 16 vials (IQR 8–34), giving an approximate average daily dose of 4 MIU. Both the duration of dispensing and the cumulative quantity were greater among non-survivors than among survivors (median 5 versus 4 days, and 21 versus 15 vials, respectively; both *p* < 0.001; Table 1). Length of stay was also longer among non-survivors than among survivors (median 16 (IQR 9–27) vs. 12 (IQR 7–19) days, respectively; *p* < 0.001).

### 2.2. In-Hospital Mortality

In-hospital mortality was 27.6% (338/1225). In unadjusted comparisons, non-survivors were significantly older than survivors (68.4 ± 12.8 versus 63.8 ± 14.7 years; *p* < 0.001) and were more frequently admitted via the emergency route (76.0% versus 56.1%; *p* < 0.001). Acute kidney injury was markedly more prevalent among non-survivors than survivors (46.7% versus 17.9%; *p* < 0.001), as was sepsis (62.4% versus 31.5%; *p* < 0.001). A higher Charlson comorbidity index, diabetes mellitus, congestive heart failure, and cerebrovascular disease were also significantly associated with death (all *p* < 0.001). Neither chronic kidney disease nor end-stage renal disease (*p* = 0.608) nor male sex (*p* = 0.275) was associated with in-hospital mortality in unadjusted analysis.

### 2.3. Independent Predictors of In-Hospital Mortality

**Figure 1:**
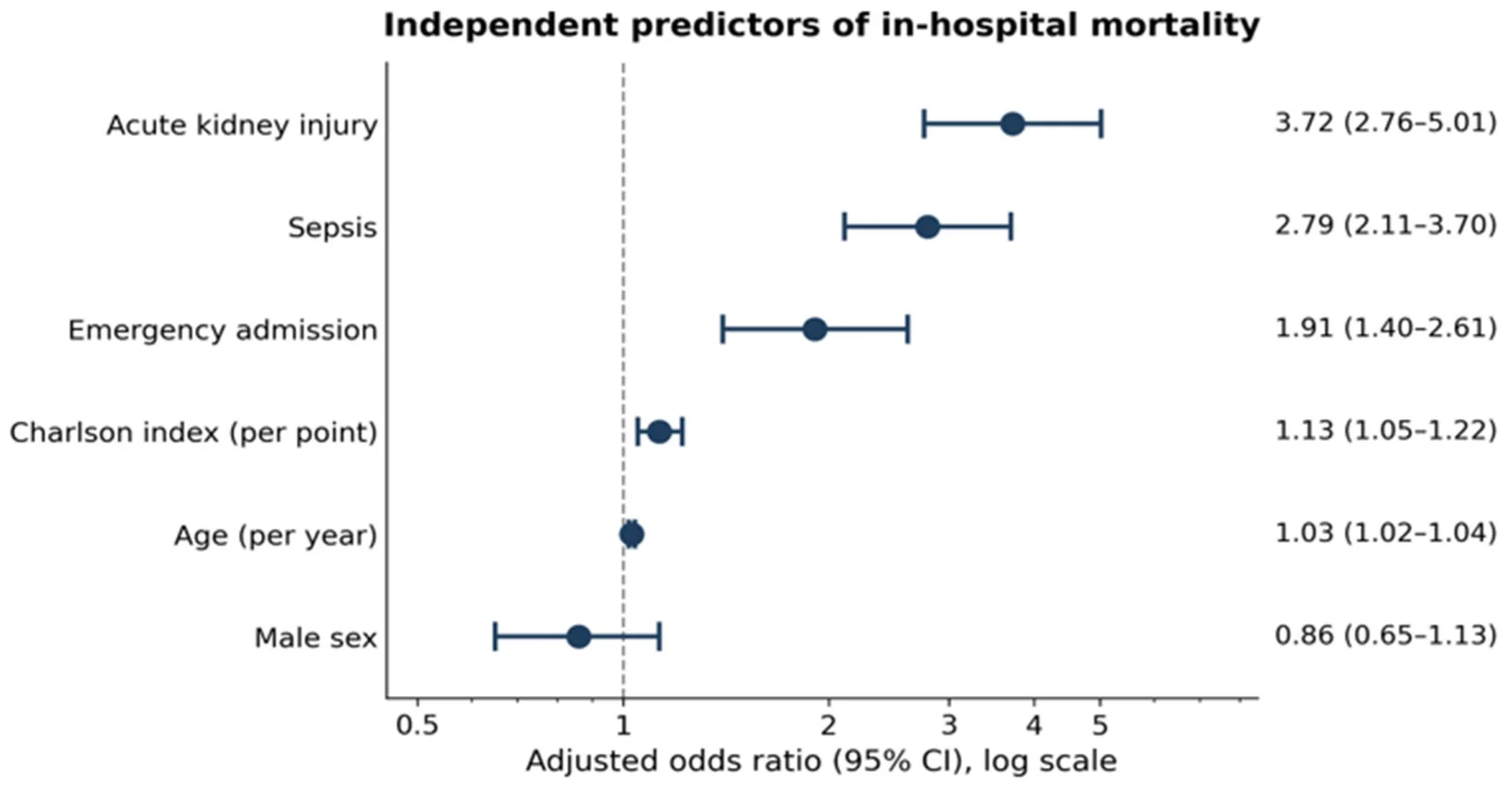
Forest plot of the multivariable logistic regression model for in-hospital mortality. Adjusted odds ratios (aORs) and 95% confidence intervals are shown for age (per year), male sex (vs. female), emergency admission (vs. elective), Charlson comorbidity index (per point), acute kidney injury, and sepsis. The dashed vertical line indicates the line of no effect (adjusted odds ratio = 1.0); estimates to the right of the line indicate increased odds of in-hospital death and estimates to the left indicate decreased odds. The x-axis is shown on a logarithmic scale. All six variables were entered simultaneously. Colistin duration, cumulative colistin quantity, and length of stay were not included in the primary model because they accrue during hospitalization and may be influenced by the subsequent clinical course and outcome.

The primary multivariable logistic regression model included six prespecified covariates: age, sex, emergency admission, Charlson comorbidity index, acute kidney injury, and sepsis. All six variables were entered simultaneously. Colistin duration, cumulative colistin quantity, and length of stay were not included in the primary model because they accrue during hospitalization. The results of the primary model are presented in Table 2 and Figure 1.

Acute kidney injury was the strongest predictor (adjusted odds ratio [aOR] 3.72, 95% confidence interval [CI] 2.76–5.01), followed by sepsis (aOR 2.79, 95% CI 2.11–3.70) and emergency admission (aOR 1.91, 95% CI 1.40–2.61). The odds of death increased with each additional point of the Charlson comorbidity index (aOR 1.13, 95% CI 1.05–1.22) and each additional year of age (aOR 1.03, 95% CI 1.02–1.04). Male sex was not independently associated with in-hospital mortality (aOR 0.86, 95% CI 0.65–1.13; *p* = 0.279). Variables accrued after the initiation of colistin treatment—duration, cumulative dose, and duration of hospitalization—were not entered into the model. The model comprised six predictors for 338 events (approximately 56 events per variable) and was significant overall (χ^2^ = 227.7, df = 6, *p* < 0.001; Nagelkerke R^2^ = 0.245). Discrimination was acceptable, with an area under the receiver operating characteristic curve of 0.773 (95% CI 0.748–0.796), and 74.0% of cases were correctly classified. The Hosmer–Lemeshow test indicated a departure from ideal calibration ($\chi^{2}=18.57,\;df=8,\;p=0.017$), inspection of the decile table showed over-prediction of mortality in the highest-risk decile and under-prediction in the middle deciles. Although the multivariable model demonstrated acceptable discrimination (AUC of 0.773), this departure shows a tendency to overpredict risk in the highest-risk decile; therefore, our model should be viewed exclusively as a tool for relative risk ranking at the population level rather than as an absolute survival predictor applicable to an individual patient at the bedside.

### 2.4. Changes in In-Hospital Mortality over Time

Temporal analyses were conducted separately from the primary multivariable mortality model described above. Calendar year of admission was analyzed as a continuous predictor to assess the annual change in mortality. In the crude model, the odds of in-hospital death increased by approximately 16% per year (OR 1.162, 95% CI 1.126–1.199; *p* < 0.001). After adjustment for the covariates included in the primary model, the association remained significant (aOR 1.129 per year, 95% CI 1.089–1.169; *p* < 0.001).

**Figure 2:**
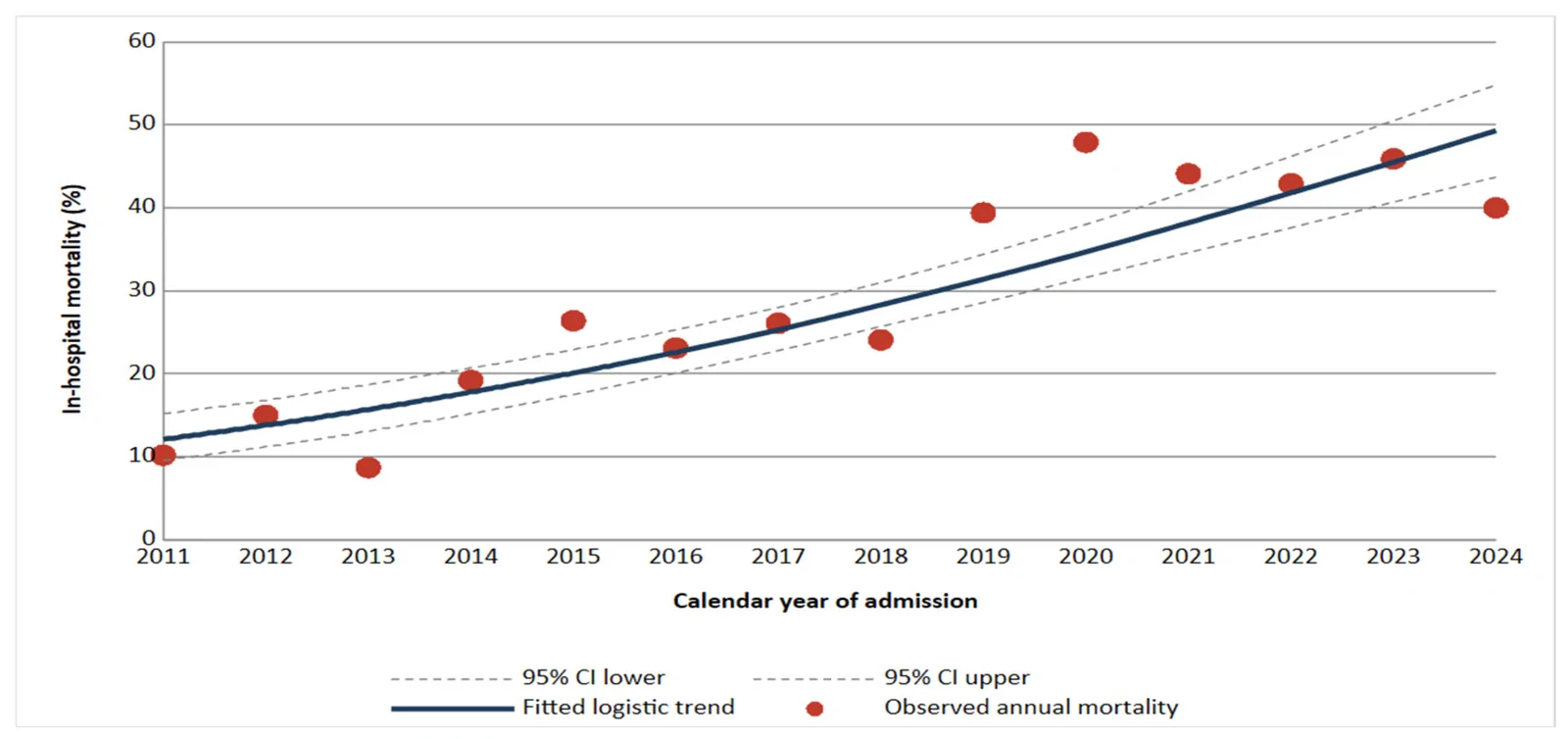
Temporal trend in in-hospital mortality by calendar year of admission among colistin-treated patients (*n* = 1217). The fitted probability and its 95% confidence interval are derived from a univariable logistic regression model with calendar year entered as a continuous predictor (OR 1.162 per year, 95% CI 1.126–1.199; *p* < 0.001). Calendar years 2009 and 2010, comprising eight patients in total, were excluded because the small number of admissions precluded stable annual estimates.

Over the study period, in-hospital mortality among colistin-treated patients increased substantially (Figure 2). Annual mortality rose from 10.2% in 2011 to 40.0% in 2024, ranging between 8.7% and 19.2% during 2011–2014 and between 42.9% and 47.9% during 2020–2023. In a logistic regression model with calendar year of admission entered as a continuous predictor, the crude odds of in-hospital death increased by approximately 16% per year (odds ratio 1.162, 95% CI 1.126–1.199; *p* < 0.001). After adjustment for age, sex, emergency admission, Charlson comorbidity index, acute kidney injury, and sepsis, the association was attenuated but remained highly significant (adjusted odds ratio 1.129 per year, 95% CI 1.089–1.169; *p* < 0.001), indicating that changing case-mix accounted for only part of the trend. When the cohort was dichotomized at the midpoint of the study period, mortality was more than twofold higher in the second half than in the first (38.6% [234/606] in 2017–2024 versus 16.8% [104/619] in 2009–2016; *p* < 0.001). Over the same period, the proportion of patients presenting with sepsis rose from 28.2% in 2011–2013 to 53.0% in 2022–2024, acute kidney injury from 18.2% to 27.4%, emergency admission from 53.4% to 72.6%, and the mean Charlson comorbidity index from 2.30 to 3.25. Excluding the pandemic years 2020–2021 did not materially alter the annual increase (odds ratio 1.155 per year, 95% CI 1.118–1.193). The two earliest calendar years (2009–2010) together comprised only eight patients and were excluded from the trend analysis to avoid unstable annual estimates. Data capture for 2024 extended only until 25 November.

## 3. Discussion

Colistin occupies a paradoxical position in contemporary practice: an antibiotic of historical vintage and appreciable toxicity that has become indispensable precisely where therapeutic options are fewest. In high-resistance settings such as Romania, where it is deployed outside the intensive-care unit to a broad population of complex inpatients, understanding which patients die while receiving colistin, and how this has changed as the resistance landscape and prescribing practices has evolved, is a prerequisite for the rational use of a last-line agent. The present 15-year cohort was designed to address these questions in a setting under-represented in the existing literature.

In this 15-year retrospective cohort of 1225 patients receiving systemic colistin at a tertiary urology–nephrology center, more than one in four died before discharge. Five characteristics ascertainable at the moment colistin was initiated—acute kidney injury, sepsis, emergency admission, comorbidity burden, and age—were independently associated with in-hospital death, and together discriminated survivors from non-survivors with an area under the receiver operating characteristic curve of 0.77. Sex was not associated with outcome. Mortality increased markedly across the study period, from 10.2% in 2011 to 40.0% in 2024. Adjustment for case-mix attenuated this by only about one-fifth, leaving much of the change unexplained by the patient characteristics we were able to measure.

The observed in-hospital mortality of 27.6% is lower than the approximately 44% all-cause 28-day mortality and 50% intensive care mortality reported in cohorts treated for carbapenem-resistant Gram-negative infection [16,17]. This difference is most plausibly attributable to case-mix rather than to superior care: our cohort was not restricted to critically ill patients, and comprised predominantly ward-based urology and nephrology admissions in a center where colistin is frequently prescribed for complicated urinary tract and catheter-associated infection. This population is infection-prone through instrumentation, obstructive uropathy, and post-transplant immunosuppression, yet is largely managed outside the intensive-care unit, and it is under-represented in the existing literature [24,26,30]. The prognostic factors we identified nevertheless overlap substantially with those reported from intensive-care and mixed cohorts, in which older age, greater Charlson comorbidity burden, and renal impairment have each been independently associated with death [22,23,30]. That the same determinants operate outside the intensive-care setting, and at a lower absolute level of risk, suggests that they reflect general host vulnerability rather than features specific to critical illness.

Acute kidney injury showed the strongest adjusted association with in-hospital death, corresponding to an almost fourfold increase in the odds of mortality; ascertained from discharge codes without creatinine trajectories, this finding identifies a prognostic marker rather than a mechanism.

This is consistent with the narrow therapeutic index of colistin and with pooled estimates indicating that a substantial proportion of treated patients develop nephrotoxicity [11,12,13,14,15]. Two interpretations are not mutually exclusive. Acute kidney injury may act as a marker of systemic illness severity, integrating haemodynamic compromise, sepsis, and pre-existing renal vulnerability into a single readily available variable. Alternatively, or additionally, it may represent the clinical expression of colistin nephrotoxicity itself, which is dose-dependent and particularly consequential in patients whose renal reserve is already diminished—as it was in the 46.4% of our cohort with chronic kidney disease or end-stage renal disease [19,20,31,32]. Our data cannot separate these mechanisms: we recorded acute kidney injury from coded diagnoses without a serum creatinine trajectory, and could not attribute individual episodes to colistin as opposed to sepsis, obstruction, contrast exposure, or concomitant nephrotoxic drugs. What the finding does establish is that renal dysfunction identified around the time of colistin initiation carries substantial prognostic weight in this population and should prompt heightened vigilance rather than reassurance.

An alternative explanation for the temporal increase in mortality would be a change in institutional colistin prescribing practices, such as implementation of an antimicrobial stewardship intervention or restriction of colistin to patients with more severe infections. However, review of the available institutional prescribing protocols and administrative records did not identify any major policy change or specific restriction affecting colistin use during the study period. Nevertheless, because this was a retrospective study, we cannot exclude more subtle changes in prescribing behavior, indications for colistin use, or other unmeasured changes in clinical practice that may have occurred over time.

Dosing practices also merit consideration as a potential contributor to both the observed outcomes and the temporal trend. The approximate mean daily dose in our cohort (approximately 4 MIU) was lower than the maintenance dose of approximately 9 MIU/day, as recommended by the International Consensus Guidelines for patients with preserved renal function [6]. However, given the high prevalence of renal impairment in our cohort, this comparison should not be interpreted as evidence of systematic underdosing. Colistimethate sodium is a prodrug that undergoes slow and variable conversion to active colistin, and dosing regimens based solely on body weight and creatinine clearance, as recommended in older guidance, are now recognized to result in subtherapeutic exposure in a substantial proportion of patients while providing only limited protection against nephrotoxicity [6,11]. The short median treatment duration of four days was likewise shorter than the conventional course for multidrug-resistant Gram-negative infections and likely reflects a combination of early mortality, de-escalation following microbiological results, and discontinuation of empirical therapy. Consequently, suboptimal drug exposure and abbreviated treatment duration may have contributed to therapeutic failure independently of pathogen resistance. However, because data on body weight, renal function at treatment initiation, loading dose administration, dose adjustments, and plasma colistin concentrations were unavailable, this interpretation remains speculative. Prospective studies incorporating detailed pharmacokinetic data and therapeutic drug monitoring are needed to clarify the contribution of dosing optimization to clinical outcomes.

Sepsis and emergency admission were the next strongest predictors, together describing the acuity of the presenting illness. The longer length of stay observed among non-survivors also warrants consideration. In this retrospective cohort, length of stay should not be interpreted as a baseline characteristic or as a cause of mortality. Rather, the longer stay among non-survivors may reflect the greater clinical complexity and disease burden observed in this group, including the higher prevalence of comorbidities, sepsis, acute kidney injury, and emergency admission. At the same time, length of stay is intrinsically influenced by the clinical course and survival: patients who survive longer have more opportunity to accumulate hospital days, whereas early death necessarily truncates length of stay. Therefore, the observed longer stay among non-survivors likely reflects a combination of greater illness complexity and the evolving clinical trajectory rather than an independent effect of hospitalization duration on mortality.

Sepsis nearly trebled the adjusted odds of death, and admission via the emergency route almost doubled them, the latter plausibly serving as a proxy for unplanned clinical deterioration, later presentation, and less opportunity for pre-admission optimization. Comorbidity burden and age contributed more modestly on a per-unit basis, 13% higher adjusted odds per additional Charlson point and 3% per additional year, but across the observed range of these variables their cumulative contribution was considerable [22,23,26]. Notably, chronic kidney disease or end-stage renal disease was not associated with mortality in unadjusted analysis, in contrast to acute kidney injury; in a nephrology-dense cohort, stable chronic renal impairment appears to behave differently from acute renal decompensation, and the two should not be conflated in risk assessment [25].

The progressive increase in mortality across the study period is, in our view, the most consequential and least straightforward of our findings. Case-mix demonstrably worsened: between 2011–2013 and 2022–2024, the proportion of patients presenting with sepsis rose from 28.2% to 53.0%, emergency admissions from 53.4% to 72.6%, acute kidney injury from 18.2% to 27.4%, and the mean Charlson comorbidity index from 2.30 to 3.25. One hypothesis, which our data cannot directly test in the absence of microbiological information, is indication drift. Over the same interval, newer agents active against carbapenem-resistant Gram-negative organisms entered practice and antimicrobial stewardship programs matured [33,34], progressively confining colistin to patients with the most resistant isolates and the fewest therapeutic alternatives. On this account, rising mortality among colistin recipients reflects a narrowing and increasingly severe indication rather than deteriorating quality of care; however, without pathogen identity, resistance phenotype, and colistin-susceptibility data, this interpretation must remain conjectural.

Critically, adjustment for the measured case-mix reduced the annual increase only from an odds ratio of 1.162 to 1.129 per year, so roughly four-fifths of the trend remained unexplained. Residual confounding by unmeasured severity is likely, but the magnitude of the residual also raises the possibility of factors our dataset could not capture: increasing carbapenem resistance and pathogen difficulty, greater frequency of pandrug-resistant isolates, delays to effective therapy, or reduced susceptibility to colistin itself [35,36,37]. The trend was not an artefact of the COVID-19 period, since excluding 2020–2021 left it essentially unchanged [38,39,40]. These observations should be regarded as hypothesis-generating and as a direct argument for prospective surveillance linking colistin use to microbiological and susceptibility data. Beyond measured case-mix, the 15-year window encompassed profound secular changes in practice that plausibly shaped both the selection of patients for colistin and their outcomes. Diagnostic capability improved, with broader access to automated identification and susceptibility testing and, latterly, molecular detection of carbapenemases, shortening the time to recognition of resistant infection but also channeling the most resistant cases toward colistin. Novel agents active against carbapenem-resistant organisms, including ceftazidime–avibactam and subsequently cefiderocol, entered Romanian practice during the second half of the study period, progressively displacing colistin toward salvage indications [33,34]. Antimicrobial stewardship and infection-control programs matured under evolving national regulation; although we identified no formal institutional restriction of colistin during the study period, national policy and prescribing culture around last-line agents changed appreciably. The COVID-19 pandemic transiently altered case-mix, hospital operations, and resistance ecology, although excluding 2020–2021 left the trend essentially unchanged [38,39,40]. Each of these forces operates in the direction of concentrating colistin use in progressively sicker patients with fewer alternatives, and none is captured by the covariates available to us; the residual temporal trend should be read with this in mind.

This study has several strengths. It covers 15 consecutive years and, to our knowledge, represents one of the larger single-center cohorts of colistin-treated inpatients reported outside the intensive-care setting. Inclusion was consecutive and unselected, comorbidities were derived from ICD-10 codes using a published algorithm rather than from unstructured text, and the analysis was restricted to one admission per patient so that observations were independent. The multivariable model included six predictors for 338 events, well above conventional thresholds for overfitting. Importantly, we prespecified a primary model based on demographic, admission-related, comorbidity, acute kidney injury, and sepsis variables, while excluding colistin duration, cumulative dose, and length of stay because these variables accrue during hospitalization and may be influenced by the subsequent clinical course and outcome. Several limitations must be acknowledged. The study is retrospective and confined to a single tertiary center with a specialty-specific case-mix, which limits generalizability. We lacked microbiological data on pathogen identity, carbapenem-resistance phenotype, colistin susceptibility, and infection site, which is the most important gap, since these variables plausibly explain much of the unexplained temporal trend. A major limitation of our study is the absence of detailed microbiological data regarding pathogen identity, carbapenem-resistance phenotypes, infection site, and colistin susceptibility. Although we hypothesized that the temporal increase in mortality reflects an ‘indication drift’ (the gradual restriction of colistin to the most severe patients due to the introduction of newer antimicrobial agents), the lack of these data prevents us from directly quantifying the impact of bacteriology. Therefore, the observed temporal trend must be interpreted with caution and validated in future studies correlating colistin exposure with resistance profiles. No validated severity score such as SOFA or APACHE II was available, nor an indicator of intensive-care admission, so residual confounding by illness severity is probable, and the adjusted temporal trend should be interpreted with corresponding caution. We had no data on dosing appropriateness, use of a loading dose, combination versus monotherapy, or therapeutic drug monitoring, all of which influence both efficacy and nephrotoxicity [18,19,20,21,31]. Finally, our study lacks essential pharmacokinetic information, such as dosing appropriateness, use of loading doses, combination versus monotherapy regimens, or therapeutic drug monitoring (TDM) factors that significantly influence both renal safety profiles and clinical efficacy in colistin treatment. Diagnoses were ascertained from coded discharge data, which is subject to misclassification and may under-record conditions not documented at discharge. Importantly, we could not establish the timing of acute kidney injury relative to the initiation of colistin; renal dysfunction present at the start of therapy and renal dysfunction emerging during it carry different interpretations, and our data do not distinguish them. An important aspect to note is that we were unable to establish the exact timing of acute kidney injury (AKI) relative to the initiation of colistin therapy due to the absence of daily serum creatinine trajectories in the coded data. Consequently, AKI captured via discharge codes acts primarily as a strong marker of systemic severity and baseline vulnerability, without allowing a definitive separation between colistin-induced nephrotoxicity and renal injury caused by sepsis, hemodynamic instability, or underlying renal comorbidities. Model calibration was imperfect, with evidence of over-prediction of risk in the highest-risk decile; the model should therefore be regarded as a tool for ranking relative risk rather than for estimating the absolute probability of death in an individual patient. Sixty-nine patients (4.9%) were transferred to other hospitals, and 19 were discharged against medical advice, so their ultimate outcomes are unknown; in-hospital mortality also does not capture deaths occurring after discharge, and true 30-day or 90-day mortality is likely higher than reported here. Finally, data capture for 2024 extended only to 25 November and included only discharged patients, which may under-represent long-stay and therefore higher-risk admissions in that year.

These findings have practical implications for the care of patients in whom colistin is being considered. The three characteristics with the strongest adjusted associations, acute kidney injury, sepsis, and emergency admission, are all evident at the bedside on the day therapy is started, require no additional testing, and identify a group at markedly elevated risk of death. In such patients, early involvement of critical care, meticulous renal-function-adjusted dosing with consideration of therapeutic drug monitoring, where available, active review of alternative or combination regimens, and prompt source control are all reasonable priorities [6,33]. Conversely, the absence of these features identifies a lower-risk group in whom ward-based management may be appropriate. Our model is intended as an aid to risk stratification rather than a treatment algorithm, and requires external validation in independent cohorts, ideally linked to microbiological data, before it can be recommended for routine use.

## 4. Materials and Methods

### 4.1. Study Design and Setting

We conducted a retrospective observational cohort study at “Dr. C. I. Parhon” Clinical Hospital, a 206-bed tertiary referral center for urology and nephrology in Iaşi, Romania, serving the north-eastern region of Romania. The hospital provides inpatient urological surgery, general nephrology, chronic haemodialysis, and renal transplantation services. The study period extended from November 2009, corresponding to the earliest recorded administration of systemic colistin in the institutional pharmacy database, to 25 November 2024, the date of data extraction. The study is reported in accordance with the STROBE statement for observational studies and the RECORD extension for studies conducted using routinely collected health data [41,42].

### 4.2. Participants

Eligible for inclusion were every hospitalization during which at least one dose of systemic (intravenous) colistimethate sodium was dispensed. Cases were identified by querying the institutional pharmacy dispensing database for colistimethate sodium and were linked to the corresponding hospital discharge records. No exclusions were applied on the basis of age, admitting ward, infection site, indication, baseline renal function, or duration of therapy; inclusion was consecutive and unselected. Patients receiving inhaled colistin without concomitant systemic administration were not captured by the pharmacy query and are therefore not represented in the cohort. One patient was aged 17 years at admission; all others were adults.

A total of 1409 eligible hospitalizations were identified, corresponding to 1225 unique patients. Because 121 patients contributed more than one colistin-treated admission (maximum 15), and repeated admissions within the same patient are not statistically independent, the first colistin-treated admission of each patient was designated the index admission and used for the primary analysis.

### 4.3. Data Sources and Extraction

Data were extracted from the hospital information system in a single structured export comprising, for each admission: admitting ward, medical record number, patient identifiers, date of birth, sex, age at admission, admission type, discharge type, discharge status, admission and discharge dates, hospitalization days, attending physician, principal discharge diagnosis, secondary discharge diagnoses, discharge summary text, individual colistin dispensing records with dates and quantities, cumulative colistin quantity, and number of days on which colistin was dispensed. Extraction was performed by the hospital informatics department and reviewed by the principal investigators. Diagnoses were recorded using the International Classification of Diseases, Tenth Revision (ICD-10). The dataset was pseudonymised before analysis by replacing direct identifiers with a numeric study code. Manual chart review and validation of coded variables for a random subset of records were performed by the clinical research team to ensure data accuracy.

### 4.4. Variables and Definitions

Age was recorded in completed years at admission. Sex was recorded as male or female. Emergency admission was defined as admission through the emergency department or by ambulance, as coded in the admission-type field, in contrast to scheduled admission. Admitting specialty was categorised as urology or nephrology (including renal transplantation and day-case urology and nephrology units) versus other specialties.

Comorbidities were derived from the principal and secondary ICD-10 discharge diagnosis codes of the index admission. The following code groups were applied: sepsis, A40–A41, R57.2, and R65.2 [43]; acute kidney injury, N17; chronic kidney disease or end-stage renal disease, N18, N19, Z49, and Z99.2; diabetes mellitus, E10–E14; congestive heart failure, I50, I11.0, I13.0, I13.2, I09.9, I25.5, I42, and I43; chronic obstructive pulmonary disease, J43 and J44; cerebrovascular disease, I60–I69, G45, and G46; and malignancy, C00–C97.

The Charlson comorbidity index was computed from ICD-10 codes using the algorithm of Quan et al. [44], applying the standard hierarchical rules whereby diabetes with complications supersedes uncomplicated diabetes, metastatic solid tumour supersedes localised malignancy, and severe liver disease supersedes mild liver disease. The unweighted-for-age version of the index was used, since age was entered separately as a continuous covariate. The Charlson Comorbidity Index (CCI) is an ordinal measure of comorbidity burden, with higher scores indicating a greater burden of comorbid conditions and a higher expected risk of mortality.

Colistin exposure was characterised by the number of days on which colistin was dispensed and by the cumulative quantity dispensed, expressed in vials of colistimethate sodium (1,000,000 International Units per vial). Because dispensing records may contain gaps, the number of dispensing days does not necessarily represent an uninterrupted treatment course. Admissions with a recorded cumulative quantity of zero were retained as single-dose or short-course dispensing artifacts where applicable. Length of stay was calculated as the interval in days between admission and discharge. Colistin duration, cumulative quantity, and length of stay accrue during the hospitalization and are partly determined by the outcome; accordingly, these variables were reported descriptively but were not entered into the multivariable model.

An approximate average daily dose was derived as the cumulative quantity dispensed divided by the number of dispensing days, expressed in million international units (MIU) of colistimethate sodium per day (1 MIU ≈ 33.3 mg colistin base activity). Because dispensing records reflect pharmacy issue rather than verified administration, and because neither body weight nor serum creatinine at the time of prescribing was available, doses could not be expressed as colistin base activity per kilogram or related to the average steady-state plasma concentration recommended by the International Consensus Guidelines [6]. Loading doses could not be distinguished from maintenance doses, and therapeutic drug monitoring was not available during the study period.

### 4.5. Outcome

The outcome was all-cause in-hospital mortality, defined as a discharge type of death recorded in the hospital discharge record. Patients transferred to another hospital (*n* = 69) or discharged against medical advice (*n* = 19) were retained in the cohort and classified as not having died in hospital; because their subsequent outcome was unknown, this may lead to slight underestimation of true mortality. Deaths occurring after discharge were not ascertained. As a secondary assessment, institutional prescribing protocols and available administrative records were reviewed to identify any major changes in antimicrobial stewardship policies or administrative restrictions specifically affecting colistin use during the study period.

### 4.6. Statistical Analysis

All analyses were performed using MedCalc Statistical Software version 23.5.6 (MedCalc Software Ltd., Ostend, Belgium). Continuous variables are presented as mean ± standard deviation or as median with interquartile range according to distribution, assessed by the D’Agostino–Pearson test for normality; categorical variables are presented as counts and percentages. Between-group comparisons used the Mann–Whitney U test for continuous variables and the chi-squared test for categorical variables. Independent predictors of in-hospital mortality were identified using a prespecified primary multivariable logistic regression model. The primary model included age, sex, emergency admission, Charlson comorbidity index, acute kidney injury, and sepsis, with all variables entered simultaneously. Colistin duration, cumulative colistin quantity, and length of stay were not included in the primary multivariable model because they accrue during hospitalization and may be influenced by the clinical course and outcome. These treatment- and hospitalization-related variables were therefore reported descriptively but were not considered baseline predictors of mortality. Results are reported as adjusted odds ratios (aORs) with 95% confidence intervals (CIs). Model discrimination was assessed using the area under the receiver operating characteristic curve, and calibration was assessed using the Hosmer–Lemeshow goodness-of-fit test.

Temporal trends in mortality were assessed separately from the primary mortality model. Calendar year of admission was entered as a continuous predictor in an unadjusted logistic regression model and subsequently in a model adjusted for the covariates included in the primary model. A binary early-versus-late study-period comparison was additionally performed as a sensitivity analysis.

Colistin duration was not included in the primary mortality model because it is accrued during hospitalization and is inherently dependent on the clinical course. In particular, patients who die early cannot accumulate additional treatment days, making the observed treatment duration potentially dependent on survival time. A conventional logistic regression model using total observed treatment duration would therefore be difficult to interpret as an independent association with mortality and could introduce time-dependent or reverse-causation bias. A dedicated time-dependent analysis would require longitudinal information on the timing of colistin initiation and discontinuation, clinical events, and mortality that was not available in the present dataset.

Results are reported as adjusted odds ratios with 95% confidence intervals. Model calibration was assessed by the Hosmer–Lemeshow goodness-of-fit test and discrimination by the area under the receiver operating characteristic curve. Temporal trend in in-hospital mortality was assessed by logistic regression with calendar year of admission entered as a continuous predictor, unadjusted and subsequently adjusted for the covariates of the primary model; the two earliest calendar years, 2009 and 2010, together comprised eight patients and were excluded from this analysis. All tests were two-sided, and *p* < 0.05 was considered statistically significant.

No major institutional change in colistin stewardship or prescribing restrictions was identified during the study period; however, changes in pathogen ecology, indications for colistin use, prescribing behavior, or other unmeasured factors may have contributed to the temporal trend. Early identification of colistin recipients presenting with acute renal dysfunction and sepsis offers a simple, immediately available basis for risk stratification in this vulnerable population. 

### 4.7. Ethical Considerations

The study was conducted in accordance with the Declaration of Helsinki and was approved by the Ethics Committee of Spitalul Clinic “Dr. C. I. Parhon” Iași (approval number 7246, dated 20 July 2026). Given the retrospective design and the exclusive use of pseudonymised, routinely collected clinical data, the requirement for individual informed consent was waived by the Ethics Committee. No identifiable patient information is reported.

## 5. Conclusions

Among 1225 patients treated with systemic colistin over 15 years at a tertiary urology–nephrology center, in-hospital mortality was 27.6% and was independently predicted by acute kidney injury, sepsis, emergency admission, comorbidity burden, and age. These findings identify clinical characteristics associated with mortality but should not be interpreted as establishing causality.

Mortality increased approximately 16% per year in relative terms across the study period, and only a minority of this increase was explained by an increasingly severe case-mix; whether changing pathogen ecology and a progressively narrowing indication for colistin accounts for the remainder is a hypothesis that warrants prospective investigation linked to microbiological data.

Early identification of colistin recipients presenting with acute renal dysfunction and sepsis offers a simple, immediately available basis for risk stratification in this vulnerable population.

## Figures and Tables

**Table 1:** Baseline characteristics of colistin-treated patients, overall and stratified by in-hospital survival status.

Characteristic	All (*n* = 1225)	Survivors (*n* = 887)	Deceased (*n* = 338)	*p*-Value
Age, years (mean ± SD)	65.1 ± 14.4	63.8 ± 14.7	68.4 ± 12.8	<0.001
Male sex, *n* (%)	737 (60.2)	542 (61.1)	195 (57.7)	0.275
Emergency admission, *n* (%)	755 (61.6)	498 (56.1)	257 (76.0)	<0.001
Urology/Nephrology ward, *n* (%)	1049 (85.6)	746 (84.1)	303 (89.6)	0.013
Charlson comorbidity index, median [IQR]	3 (2–4)	2 (1–4)	3 (2–4)	<0.001
Chronic kidney disease/ESRD, *n* (%)	569 (46.4)	408 (46.0)	161 (47.6)	0.608
Acute kidney injury, *n* (%)	317 (25.9)	159 (17.9)	158 (46.7)	<0.001
Diabetes mellitus, *n* (%)	344 (28.1)	220 (24.8)	124 (36.7)	<0.001
Congestive heart failure, *n* (%)	311 (25.4)	193 (21.8)	118 (34.9)	<0.001
COPD, *n* (%)	105 (8.6)	74 (8.3)	31 (9.2)	0.643
Cerebrovascular disease, *n* (%)	130 (10.6)	75 (8.5)	55 (16.3)	<0.001
Malignancy (any), *n* (%)	265 (21.6)	208 (23.4)	57 (16.9)	0.012
Sepsis, *n* (%)	490 (40.0)	279 (31.5)	211 (62.4)	<0.001
Colistin duration, days, median [IQR] ^1^	4 (2–7)	4 (2–6)	5 (2–10)	<0.001
Colistin cumulative quantity, vials, median [IQR] ^1^	16 (8–34)	15 (7–30)	21 (10–47)	<0.001
Length of stay, days, median [IQR] ^1^	13 (8–21)	12 (7–19)	16 (9–27)	<0.001

^1^ Reported descriptively only; these variables accrued during hospitalization and were not entered into the multivariable model. SD, standard deviation; IQR, interquartile range; ESRD, end-stage renal disease; COPD, chronic obstructive pulmonary disease.

**Table 2:** Primary multivariable logistic regression model of factors independently associated with in-hospital mortality (*n* = 1225).

Predictor	aOR (95% CI)	*p*-Value
Acute kidney injury	3.72 (2.76–5.01)	<0.001
Sepsis	2.79 (2.11–3.70)	<0.001
Emergency admission	1.91 (1.40–2.61)	<0.001
Charlson comorbidity index (per point)	1.13 (1.05–1.22)	<0.001
Age (per year)	1.03 (1.02–1.04)	<0.001
Male sex (vs. female)	0.86 (0.65–1.13)	0.279

aOR, adjusted odds ratio; CI, confidence interval. All six prespecified variables were entered simultaneously. Colistin duration, cumulative colistin quantity, and length of stay were not included in the primary model. Area under the receiver operating characteristic curve: 0.773 (95% CI 0.748–0.796).

## Data Availability

The data supporting the findings of this study contain potentially identifying clinical information and patient health records from Spitalul Clinic “Dr. C. I. Parhon” Iași, and are therefore not publicly available due to privacy and ethical restrictions. De-identified data may be made available from the corresponding author on reasonable request and subject to approval by the institutional ethics committee.
